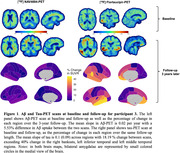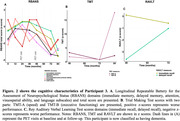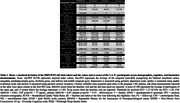# A‐T+ PET participants in preclinical AD: Clinical progression and concordance with fluid markers

**DOI:** 10.1002/alz.092369

**Published:** 2025-01-09

**Authors:** Yara Yakoub, Frédéric St‐Onge, Alfonso Fajardo, Bery Mohammediyan, Christine Dery, Elisabeth Sylvain, Jennifer Tremblay‐Mercier, Jordana Remz, Julie Gonneaud, Jacob W. Vogel, Jean‐Paul Soucy, Alexa Pichet Binette, Sylvia Villeneuve, PREVENT‐AD Research Group

**Affiliations:** ^1^ Douglas Mental Health University Institute, Centre for Studies on the Prevention of Alzheimer's Disease (StoP‐AD), Montréal, QC Canada; ^2^ Clinical Memory Research Unit, Lund University, Lund Sweden; ^3^ Montreal Neurological Institute, McGill University, Montréal, QC Canada

## Abstract

**Background:**

Amyloid‐negative tau‐positive PET (A‐T+) participants have been reported in several studies. We assessed the prevalence and characteristics of A‐T+ participants in a cohort of cognitively unimpaired individuals with a first‐degree family history of Alzheimer’s disease (AD) dementia.

**Method:**

We studied 252 participants from the longitudinal PREVENT‐AD cohort (mean cognitive follow‐up = 3.42 years, SD = 2.86) who received an amyloid (^18^F‐NAV4694) and a tau (^18^F‐Flortaucipir) PET‐scan. The amyloid‐positivity threshold was 18 centiloids (SUVR=1.27) and tau positive threshold was set as 2SD above the mean of A‐ participants in a temporal meta‐ROI (SUVR=1.29). Follow‐up PET scans were available for 109 participants. We characterized A‐T+ participants on clinical (age, sex, APOE4 status, education, depression, apathy, anxiety, sleep), cognitive (RBANS, MMSE, ECoG) and fluid (CSF, plasma) measurements.

**Result:**

In the PREVENT‐AD cohort, only 3 (1.2 %) of all studied participants were classified as A‐T+ (see Table 1 for participants characteristics). Participants 1 and 2 were positive based on plasma Aβ_42/40_ and they had relatively low levels of tau binding that could represent early pathology. Participant 3 was also positive based on CSF and plasma, her Aβ‐PET scan was classified as positive on visual read and became quantitatively positive at follow‐up. Participant 3 had extensive unilateral tau binding at baseline that became bilateral at follow‐up (Figure 1). Figure 2 details the longitudinal cognitive trajectory of Participant 3. During the cognitive follow‐up, participants 1 developed mild cognitive impairment and participant 3 developed dementia.

**Conclusion:**

A‐T+ individuals are rare in the PREVENT‐AD cohort and the 3 A‐T+ were classified as Aβ positive based on fluid biomarkers. One of the 3 A‐T+ individuals showed a very fast clinical progression and a tau uptake pattern atypical of AD.